# Dr. John Cooper

**Published:** 1910-06-04

**Authors:** 


### Obituary.

We have to record the death of Dr. John Cooper, who was very nearly a centenarian, having reached the age of 98. Dr. Cooper was a member of the Royal College of Physicians and a licentiate of the Society of Apothecaries.

